# Organocatalytic Stereoselective Cyclic Polylactide Synthesis in Supercritical Carbon Dioxide under Plasticizing Conditions

**DOI:** 10.3390/polym10070713

**Published:** 2018-06-28

**Authors:** Nobuyuki Mase, Moniruzzaman, Shoji Yamamoto, Yoshitaka Nakaya, Kohei Sato, Tetsuo Narumi

**Affiliations:** 1 Department of Engineering, Graduate School of Integrated Science and Technology, Shizuoka University, 3-5-1 Johoku, Hamamatsu, Shizuoka 432-8561, Japan; moniruzzaman.17@shizuoka.ac.jp (M.); f0330253@ipc.shizuoka.ac.jp (S.Y.); nakaya.yoshitaka.14@shizuoka.ac.jp (Y.N.); sato.kohei@shizuoka.ac.jp (K.S.); narumi.tetsuo@shizuoka.ac.jp (T.N.); 2 Graduate School of Science and Technology, Shizuoka University, 3-5-1 Johoku, Hamamatsu, Shizuoka 432-8561, Japan; 3 Research Institute of Green Science and Technology, Shizuoka University, 3-5-1 Johoku, Hamamatsu, Shizuoka 432-8561, Japan

**Keywords:** organocatalyst, thiourea, cyclic polylactide, CO_2_ plasticizing polymerization, stereocomplexes

## Abstract

Cyclic polylactide (*c*PLA) is a structural isomer of linear polylactide (PLA) although it possesses unique functionalities in comparison to its linear counterpart. Hitherto, the control of stereochemical purity in conventional *c*PLA synthesis has not been achieved. In this study, highly stereochemically pure *c*PLA was synthesized in the absence of a metal catalyst and organic solvent, which required high consumption of the residual monomer. The synthesis was conducted in supercritical carbon dioxide under CO_2_ plasticizing polymerization conditions in the presence of an organocatalyst and thiourea additives. In comparison with the stereocomplexes synthesized through conventional methods, *c*PLA from l-lactide (*c*PLLA) and *c*PLA from d-lactide (*c*PDLA) were synthesized with higher stereochemical purity and improved thermal stability. Moreover, the method presented herein is environmentally friendly and thus, applicable on an industrial level.

## 1. Introduction

Linear polylactides (PLAs) have high biodegradability and biocompatibility, with their potential applications in the medical, pharmaceutical and materials fields having drawn a significant amount of attention [1,2,3]. Cyclic polylactide (*c*PLA), which is a structural isomer of PLA with an unique topology, also displays favorable properties [4,5,6,7,8]. For example, a study on the effect of *c*PLAs on tumor cells and tumor-bearing mice demonstrated that *c*PLAs effectively inhibit tumor cell growth [9]. Furthermore, in a recent study, *c*PLA was used as a stabilizer for palladium nanoparticles in the synthesis of a *c*PLA-clay hybrid material, which can be a recyclable catalyst, for use in the aminocarbonylation reaction of aryl halides with various amines [10]. Despite these attractive properties, *c*PLAs remain underexplored in comparison to linear PLAs both with respect to their synthesis and practical applications [10].

**Scheme 1:**
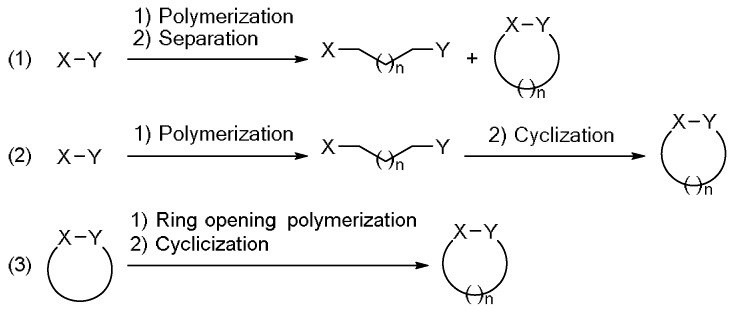
Synthetic strategies of cyclic polylactide (*c*PLA).

Until now, three strategies have been reported for the synthesis of *c*PLAs (Scheme 1): (1) polymerization and separation; (2) polymerization and cyclization; and (3) ring-opening polymerization (ROP) and cyclization. Strategy (1) has proved to be inefficient as even when fractionated by a reverse-phase octadecylsilyl (ODS) column chromatography, a mixture of linear PLAs and *c*PLAs is obtained [11]. Strategy (2) requires several steps, although uniform *c*PLAs can be eventually separated using a gel filtration column [12,13,14,15,16]. Nevertheless, it is still inappropriate for large-scale syntheses. In contrast, strategy (3) allows for a simple and straightforward one-step synthesis of *c*PLAs. The latter of these strategies was reported in 2007 using an organocatalytic method [17,18,19,20,21,22,23], while researchers have used a metal complex catalytic method in 2011 [24,25,26,27,28,29].

According to these reports, *c*PLA was obtained with high conversion (85–97%), moderate polydispersity index (PDI = 1.22–3.60) and high number-average molecular weight (Mn = 6400–69,000). The metal complex catalysts displayed poor reactivity and required high temperatures (30–160 °C) and long reaction times (4–18.5 h). In contrast, the reaction efficiency of *N*-heterocyclic carbene (NHC) as an organocatalyst was extremely high and the polymerization reaction occurred within 30 s at 25 °C. However, there were several drawbacks in that the polymerization reactions were conducted in flammable or toxic organic solvents, such as THF or CH_2_Cl_2_, and required drybox or Schlenk techniques under nitrogen, thereby limiting the potential for industrialization [19].

In general, the available methodologies fail to control the stereochemistry of PLAs, which is one of the most important parameters in material development as it has a significant effect on the properties [30,31]. In fact, in most examples of *c*PLA syntheses, racemic lactides are employed and thus, control of the stereochemical purity is insufficiently achieved. To the best of our knowledge, the stereocomplexes of *c*PLA were reported in 2011 by Waymouth et al. [19]. However, the fraction of isotactic (*iii*) tetrads of *c*PLA prepared from L-lactide was 0.81–0.83, suggesting that epimerization occurred during the polymerization step. Recently, Kricheldorf et al. reported a racemization-free polymerization, in which a cyclic tin catalyst was employed under bulk polymerization conditions, but a high temperature (160 °C) was required [29].

**Scheme 2:**
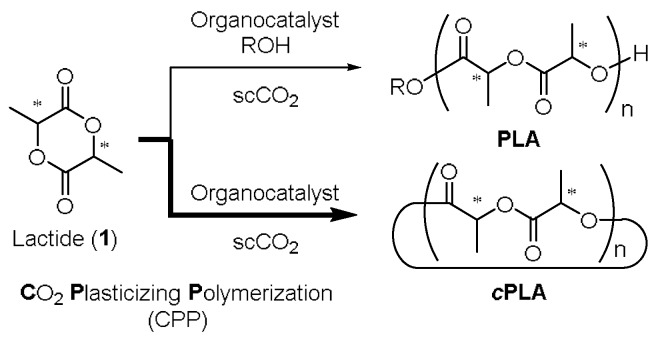
ROP of lactide, which creates PLA and *c*PLA.

Based on our previous findings on the synthesis of stereochemically pure linear PLA [32], in this study, we have developed a method for the synthesis of highly pure *c*PLA in the absence of metals, organic solvents or residual monomers. Instead, the approach employs an organocatalyst in supercritical carbon dioxide (scCO_2_) under CO_2_ plasticizing polymerization (CPP) conditions at low temperatures. The results were promising as both L- and D-*c*PLA were synthesized in high stereochemical purity from L- and D-lactide **1**, respectively (Scheme 2), which are hereafter denoted as *c*PLLA and *c*PDLA, respectively. An improvement in the thermal properties of the stereocomplexes was also observed.

## 2. Materials and Methods

### 2.1. Materials

In this study, 1-(3,5-Bis(trifluoromethyl)phenyl)-3-cyclohexyl thiourea was synthesized according to literature procedures [33]. All other chemicals and solvents were commercially available and used as received. L-Lactide was provided by Ricoh Co., Ltd. (Tokyo, Japan), while CO_2_ was obtained from Air Liquide Kogyogas Ltd. (Tokyo, Japan).

### 2.2. Synthesis

Polymerization reactions were conducted in a scCO_2_ reaction system using the TVS-N2-type portable reactor (20 MPa, 260 °C) manufactured by Taiatsu Techno Corporation (Tokyo, Japan) with the JASCO PU-1586 scCO_2_ pump (JASCO Corporation, Tokyo, Japan). L-Lactide (**1**, 864 mg, 6.0 mmol, 1.0 eq) and the organocatalyst (4-(dimethylamino)pyridine (DMAP), 0.40 mmol, 6.6 mol %) were added to a 12-mL pressure-resistant reactor, before the mixture was heated to 60 °C using a water bath. The vessel was charged with scCO_2_ (60 °C, 10 MPa) and the solution was stirred for 5 min. The reaction mixture was allowed to stand for 5 h, before the pressure was reduced from supercritical to atmospheric, yielding *c*PLA as a white solid. For the thiourea-mediated *c*PLLA synthesis, 1-(3,5-bis(trifluoromethyl)phenyl)-3-cyclohexyl thiourea (1 mol %) was added to the mixture of lactide and DMAP. This mixture was subjected to the polymerization reaction for 2.5 h as described above.

*c*PLLA and *c*PDLA samples with different number-average molecular weights were isolated by preparative gel permeation chromatography (GPC) using a recycling high performance liquid chromatography (HPLC) system (YMC LC-Forte/R, YMC CO., Ltd., Kyoto, Japan) with a YMC-GPC T30000 (21.2 mm × 600 mm) and YMC-GPC T4000 (21.2 mm × 600 mm) columns in series (CHCl_3_ as the eluent, flow rate of 6.0 mL/min). For preparation of the stereocomplex of *c*PLA [19], *c*PLLA (50 mg) and *c*PDLA (50 mg) were dissolved separately in 5 mL of CH_2_Cl_2_. These solutions were mixed together in a 20-mL flask and stirred for 5 min at room temperature. The solvent was evaporated slowly (200 torr) at room temperature, before the residue was dried under a vacuum to remove the residual solvent. The resulting material was used for DSC measurements.

### 2.3. Characterization

The conversion by polymerization was determined through ^1^H NMR analysis. ^1^H NMR (300 MHz) spectra were recorded using the JEOL JNM-AL spectrometer (JEOL, Tokyo, Japan) at ambient temperature. The spectra were recorded using CDCl_3_ as a solvent and tetramethylsilane (TMS) as an internal standard (*δ* = 0 ppm). The number- and weight-average molecular weights (Mn, Mw) and polydispersity index (PDI = Mw/Mn) of the products were determined by gel permeation chromatography (GPC) analysis using the SHIMADZU LC-5A pump (SHIMADZU, Kyoto, Japan) and JAI R1-3H RI detector (Japan Analytical Industry Co., Ltd., Tokyo, Japan) with GPC column GPC K-806L (Shodex, Tokyo, Japan, 8.0 mm × 300 mm, flow rate 0.8 mL/min), using polystyrene as a standard and CHCl_3_ as an eluent. LAsoft CDS-Lite software (LAsoft, Ltd., Chiba, Japan) was used for the molecular weight calculations.

The topology of the polymer product being cyclic rather than linear was confirmed by ^1^H NMR and matrix assisted laser desorption/ionization time-of-flight mass spectrometry (MALDI-TOF-MS) (Supplementary Materials, Figures S1 and S2). The mass spectra were recorded using ultrafleXtreme mass spectrometry (MALDI-TOF-MS, Bruker Japan, Yokohama, Japan). For MALDI-TOF-MS measurements, the *c*PLA was dissolved in CHCl_3_. *Trans*-2-[3-(4-*tert*-butylphenyl)-2-methyl-2-propenylidene]malononitrile (DCTB) was used as the matrix and silver trifluoroacetate was added as a cation source.

In order to hydrolyze the polymer materials, an aqueous solution of NaOH (1 M, 10 mL) was added to the polymer. After 4 h under reflux conditions, the mixture was cooled down to the ambient temperature and an aqueous solution of HCl (2 M, 5.5 mL) was added. The resulting lactic acid was analyzed by chiral HPLC. The HPLC analysis was conducted under the following conditions: SUMICHIRAL OA5000 column (Sumika Chemical Analysis Service, Ltd., 4.6 mm × 150 mm, flow rate 1.0 mL/min) with 2 mM CuSO_4_ aq/2-propanol = 95:5; UV detection at 254 nm (L-lactide: retention time = 8.5 min; D-lactide: retention time = 10.3 min) (Supplementary Materials, Figure S3).

Differential scanning calorimetry (DSC) analysis was performed using SHIMADZU DSC-60 (SHIMADZU, Kyoto, Japan). For DSC measurements, a 5-mg sample was heated from 25 to 250 °C at 10 °C/min rate under an argon flow (50 mL/min). The data collection interval was 1.0 s.

## 3. Results and Discussion

**Scheme 3:**
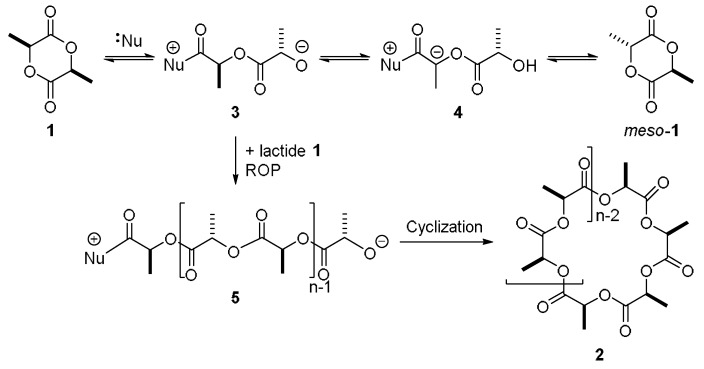
Competitive reaction between polymerization (and hence, cyclization) and epimerization.

**Figure 1:**
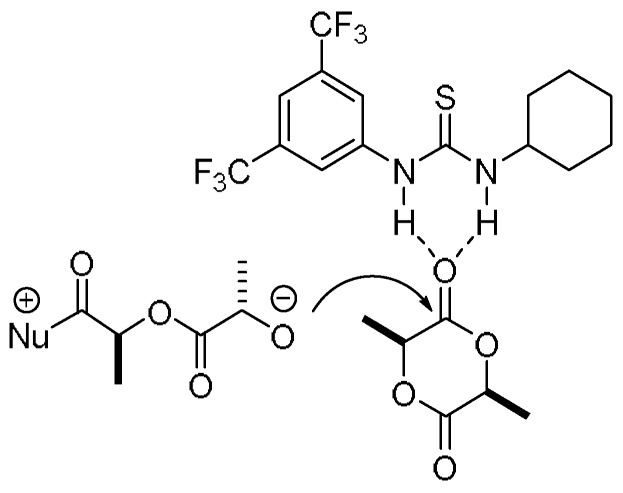
Activation of L-lactide monomer by a thiourea additive.

The synthesis of *c*PLA via an organocatalytic ROP was examined according to previous findings on the synthesis of stereochemically pure linear PLA (Table 1) [32]. Notably, ^1^H NMR analysis of the polymerization reaction mixture showed that the remaining unreacted monomers were less than 5% in any of the polymerization reactions. The monomer conversion rate and weight of the recovered polymer were both higher than 95%. In the conventional solution polymerization method, the reaction was slow and resulted in the formation of amorphous *c*PLLA with low stereochemical purity, which was determined by HPLC analysis after hydrolysis of *c*PLLA to lactic acid (Table 1, entry 1). In contrast, the reaction proceeded smoothly in scCO_2_ and crystalline *c*PLLA was obtained in a high enantiomeric excess (ee) of 90.5% (Table 1, entry 2). The success of the polymerization reaction under scCO_2_ conditions was attributed to the high concentration conditions similar to those in bulk polymerization and uniform conditions similar to those in solution polymerization. We refer to this process as a CO_2_ plasticizing polymerization (CPP) method [32]. Under CPP conditions, epimerization was suppressed since the reactive zwitterionic intermediate (**3**) was less likely to be solvated by scCO_2_ (dielectric constant (*ε*_r_) = 1.15 at 10 MPa, 60 °C) as this has a lower *ε*_r_ than pentane (*ε*_r_ = 1.84) and hexane (*ε*_r_ = 1.89) [34,35]. Thus, this allows for the ROP to be preferred over the epimerization, which would form the *meso*-lactide **1** (Scheme 3). It was assumed that the stereochemical purity of *c*PLLA could be improved by increasing the polymerization consumption rate of the monomer. Thus, the additives that selectively activated the carbonyl group of L-lactide were investigated (Figure 1). In particular, 1-(3,5-bis(trifluoromethyl)phenyl)-3-cyclohexylthiourea accelerated the ROP reaction and improved the stereochemical purity, giving an ee of 93.5% (Table 1, entry 3). Given the success of the reaction using L-lactide, the same reaction conditions were applied to the ROP of D-lactide, with *c*PDLA being obtained in high stereoselectivity (entries 4 and 5). The PDI of the *c*PLA formed herein was 1.20–1.60, which was comparable to that obtained via the previously reported NHC catalytic method (1.3–1.4) [19].

In the *c*PLA synthesis, since a cyclic structure was formed via an intramolecular cyclization, the PDI could not be improved unless the linear intermediate of the same molecular weight is selectively cyclized.

**Figure 2:**
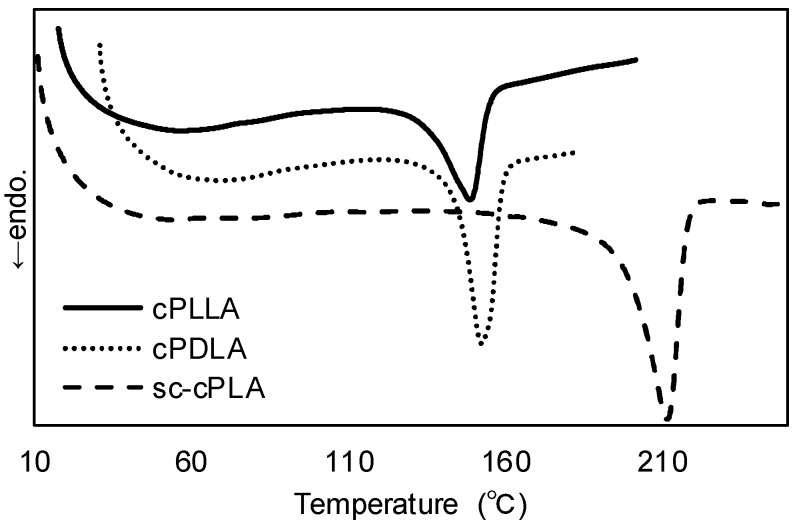
DSC measurements of the stereocomplex of *c*PLA (Table 2, entry 1).

**Figure 3:**
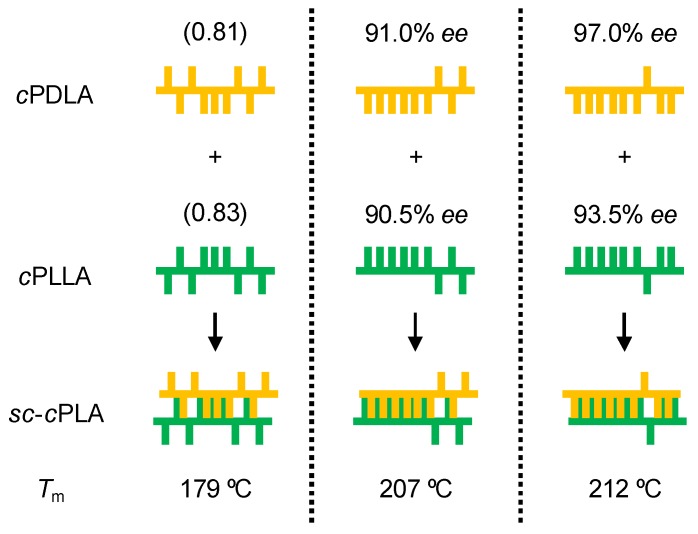
Effect of stereochemical purity on the intermolecular interactions in the stereocomplex formation.

Similar to PLA, *c*PLA has been known to form a stereocomplex of *c*PLA (sc-*c*PLA) [19,36,37]. In this work, *c*PDLA was synthesized according to the same procedure as that of *c*PLLA, while differential scanning calorimetry (DSC) was applied to create sc-*c*PLA (Figure 2). Interestingly, although the molecular weight was lower than the one reported in the literature (Table 2, entries 1 and 2 vs. 3), the measured melting point was higher than the previously reported value [19], which indicates stronger intermolecular forces. The melting point of sc-*c*PLA with the highest stereochemical purity was 212 °C. As the stereochemical purity decreased, the melting point decreased to 207 °C (Table 2, entries 1 vs. 2). Therefore, we can conclude that the higher stereochemical purity of the D- and L-forms led to stronger intermolecular interactions between the two forms in the stereocomplex and hence, a higher melting point (Figure 3). In addition, similar to the case of the stereocomplex of *c*PLA, the melting point of *c*PLA itself also depended on the stereochemical purity.

**Figure 4:**
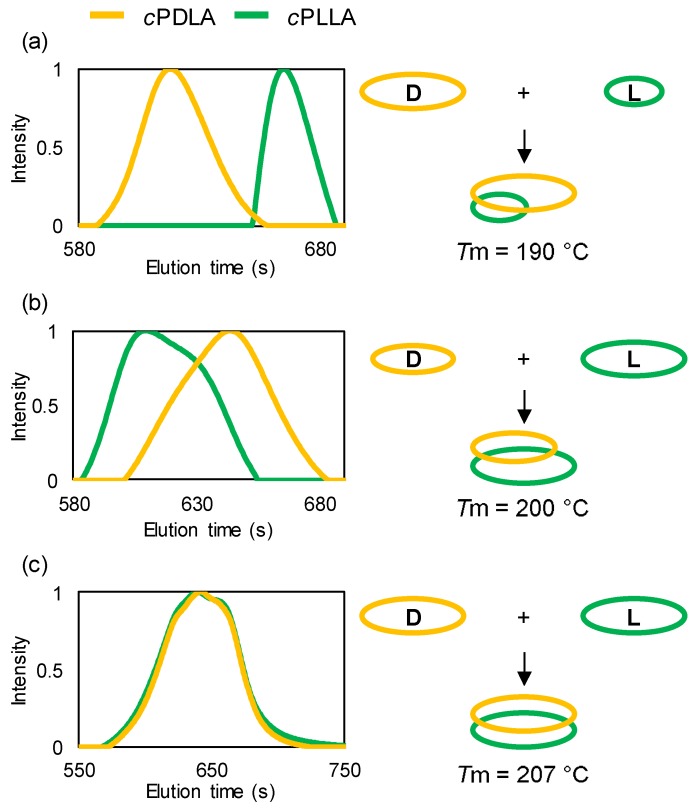
GPC traces of *c*PLA with different ring sizes and images of sc-*c*PLA.

Subsequently, the effect of the ring size on the thermal stability was investigated (Table 3 and Figure 4). Three stereocomplex samples with different number-average molecular weights were prepared using *c*PLLA and *c*PDLA, which are shown in Table 2, entry 2. These were obtained and purified by a preparative GPC. Sample 1, which had very different molecular weights of *c*PLLA and *c*PDLA, had a melting point of 190 °C (Table 3, entry 1). In Sample 2, a more moderate difference in the molecular weight distribution led to an increase in the melting point to 200 °C (Table 3, entry 2). Finally, the smallest difference in the molecular weights in sample 3 gave the highest melting point (Table 3, entry 3). These results suggested that the degree of formation of the stereocomplex depended not only on the stereochemical purity, but also on the ring size (and similarities thereof) because the overlapping portion between *c*PLLA and *c*PDLA was limited due to a different radius of the curvature.

## 4. Conclusions

In this study, highly stereochemically pure *c*PLA was synthesized in the absence of a metal catalyst or organic solvent, which required high consumption of the residual monomer. The synthesis was conducted in scCO_2_ under CO_2_ plasticizing polymerization conditions in the presence of an organocatalyst and thiourea additives. In this approach, the epimerization of the obtained *c*PLA was suppressed and the stereochemical purity was higher than those obtained from conventional methods, although the control of stereochemical purity in conventional *c*PLA synthesis has not yet been achieved. In comparison to stereocomplexes synthesized through the conventional methods, *c*PLA from L-lactide (*c*PLLA) and *c*PLA from D-lactide (*c*PDLA) stereocomplexes were synthesized with higher stereochemical purity and improved thermal stability. Based on these results, we concluded that the method presented herein could meet the increasing demands of medical and material applications in terms of safety and high functionality in the large-scale industrial manufacturing of *c*PLA and in academic research.

## Figures and Tables

**Table 1:**
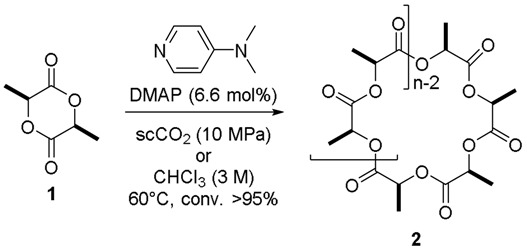
Organocatalytic *c*PLA synthesis in supercritical carbon dioxide (scCO_2_).

Entry	Solvent	Time (h)	Mn ^(a)^	PDI ^(b)^	ee (%) ^(c)^
1	CHCl_3_	120	12,700	3.11	39.0
2	scCO_2_	5	5500	1.40	90.5
3 ^(d)^	scCO_2_	2.5	11,000	1.60	93.5
4 ^(e)^	scCO_2_	5	6000	1.31	91.0
5 ^(d,e)^	scCO_2_	2.5	6800	1.20	97.0

^(a)^ Determined by GPC analysis using a polystyrene standard. ^(b)^ Polydispersity index. ^(c)^ Determined by HPLC analysis after hydrolysis of the polymer to lactic acid. ^(d)^ 1-(3,5-Bis(trifluoromethyl)phenyl)-3-cyclohexyl thiourea (1 mol%) was added. ^(e)^ d-Lactide (*ent*-**1**) was used.

**Table 2:** DSC measurements of the stereocomplex of *c*PLA.

Entry	*c*PLA	Mn	PDI	Ee (%)	*T*_m_ (°C)	*T*_m_ (sc-*c*PLA) (°C)
1	*c*PDLA	6800	1.20	97.0	152	212
	*c*PLLA	11,000	1.60	93.5	149	
2	*c*PDLA	6000	1.31	91.0	143	207
	*c*PLLA	5500	1.40	90.5	145	
3 ^(a)^	*c*PDLA	26,000	1.40	(0.81) ^(b)^	132	179
	*c*PLLA	30,000	1.30	(0.83) ^(b)^	135	

^(a)^ Data reported in reference 19. ^(b)^ Fraction of isotactic tetrads.

**Table 3:** Stereocomplex of PLA with different ring sizes.

Entry	*c*PLA	Mn	PDI	*T*_m_ (sc-*c*PLA)
1	*c*PDLA	7900	1.18	190
	*c*PLLA	2600	1.20	
2	*c*PDLA	5100	1.23	200
	*c*PLLA	7400	1.23	
3	*c*PDLA	6000	1.31	207
	*c*PLLA	5500	1.40	

## Supplementary Materials

The experimental and ^1^H NMR spectroscopic and analytic data are available online at http://www.mdpi.com/2073-4360/10/7/713/s1.
