# Supplementary material for: Organocatalytic Stereoselective Cyclic Polylactide Synthesis in Supercritical Carbon Dioxide under Plasticizing Conditions
Source: Polymers (Basel). 2018 Jun 28;10(7):713. doi: 10.3390/polym10070713 (PMC6404224; doi:10.3390/polym10070713)
Supplement: Supplementary file 1 [file polymers-10-00713-s001.pdf]

Supplementary Materials

Organocatalytic Stereoselective Cyclic Polylactide Synthesis in Supercritical Carbon Dioxide under Plasticizing Conditions

Nobuyuki Mase [1,2,3,*], Moniruzzaman [1], Shoji Yamamoto [1], Yoshitaka Nakaya [1], Kohei Sato [1] and Tetsuo Narumi [1,2,3]

[1] Department of Engineering, Graduate School of Integrated Science and Technology, Shizuoka University, 3-5-1 Johoku, Hamamatsu, Shizuoka 432-8561, Japan

[2] Graduate School of Science and Technology, Shizuoka University, 3-5-1 Johoku, Hamamatsu, Shizuoka 432-8561, Japan

[3] Research Institute of Green Science and Technology, Shizuoka University, 3-5-1 Johoku, Hamamatsu, Shizuoka 432-8561, Japan

[*] Correspondence: mase.nobuyuki@shizuoka.ac.jp; Tel.: +81-53-478-1196

moniruzzaman.17@shizuoka.ac.jp, f0330253@ipc.shizuoka.ac.jp, nakaya.yoshitaka.14@shizuoka.ac.jp, sato.kohei@shizuoka.ac.jp, narumi.tetsuo@shizuoka.ac.jp

Contents

1. Determination of the topology of polymer product 2
2. Determination of enantiomeric excess of polymers after hydrolysis 3
3. Preparative GPC to prepare different size of *c*PLA 4
4. DSC mesurements 6

1. Determination of the topology of polymer product

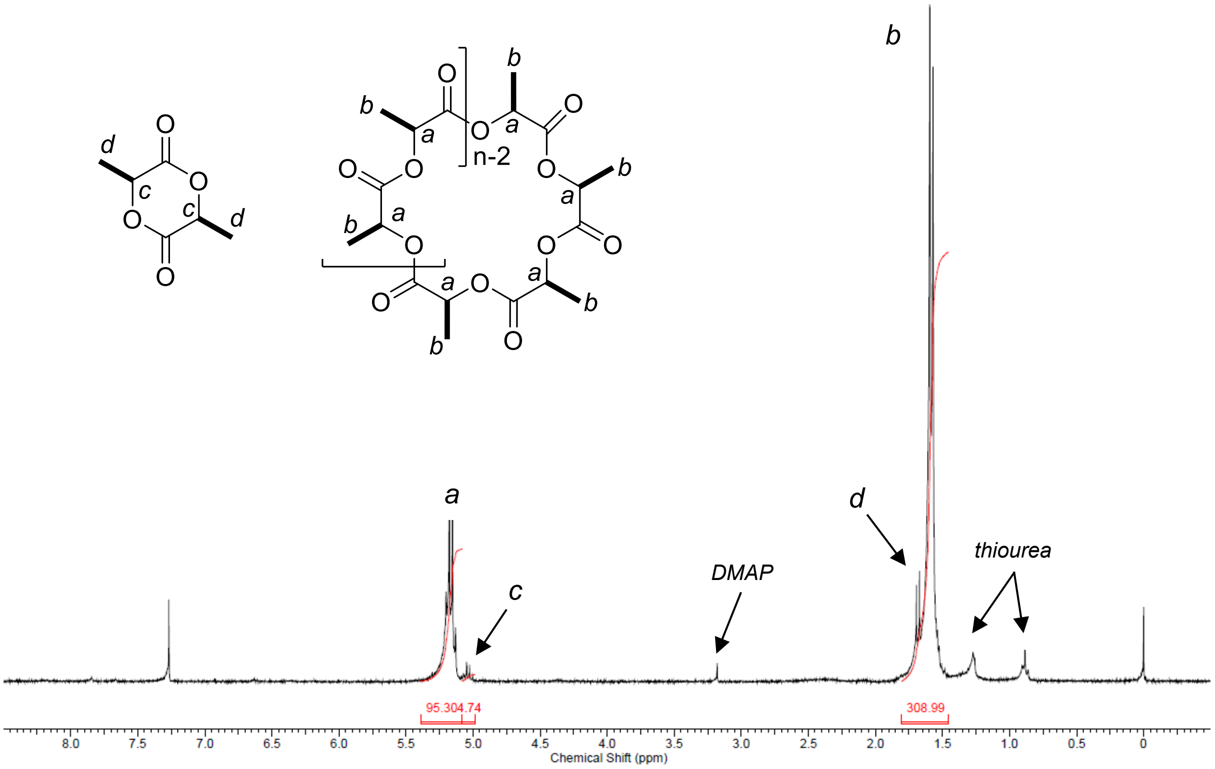

Figure S1. ^{1}H NMR spectrum of *c*PLLA synthesized by the aid of DMAP and thiourea additive.

For MALDI-TOF-MS measurement, the *c*PLLA was dissolved in $CHCl_3$; *trans*-2-[3-(4-*tert*-butylphenyl)-2-methyl-2-propenylidene]malononitrile (DCTB) was used as the matrix and silver trifluoroacetate was added as the cation source. DCTB solution in $CHCl_3$ (10 mg/mL, 9 μL), silver trifluoroacetate solution in MeOH-$CHCl_3$ (10:90 (v/v), 2 mg/mL, 2 μL) and polymer solution (1 μL) were mixed and then the mixture was spotted on a plate. The ion corresponding to [M (72.02) × n + Ag^+ (106.90)]$^+$ was detected.

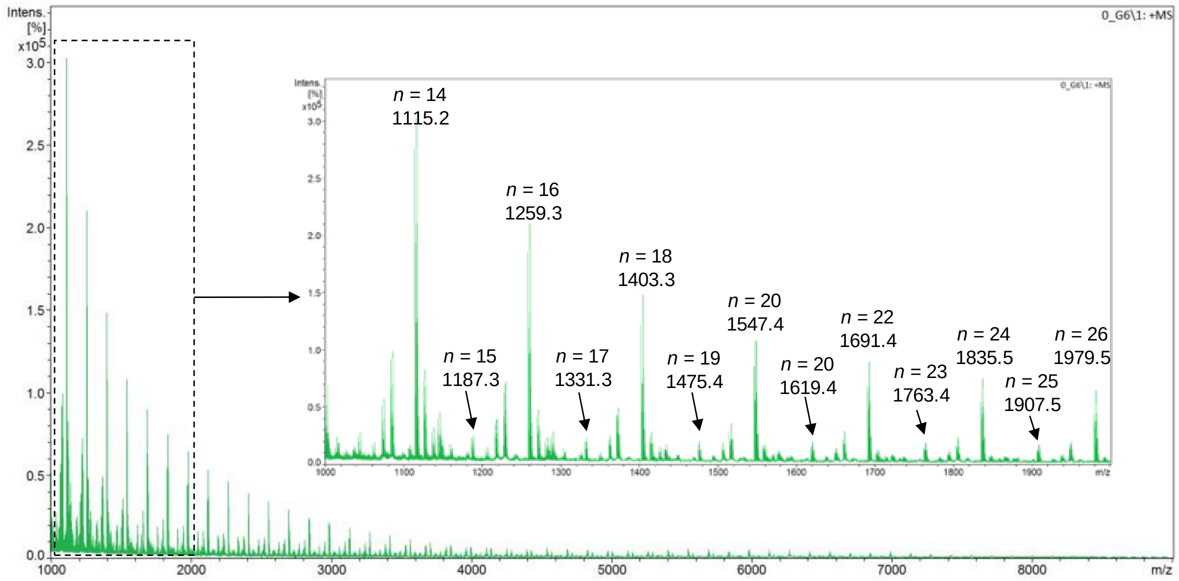

Figure S2. MALDI-TOF-MS analysis of *c*PLLA synthesized under CPP conditions.

2. Determination of enantiomeric excess of polymers after hydrolysis

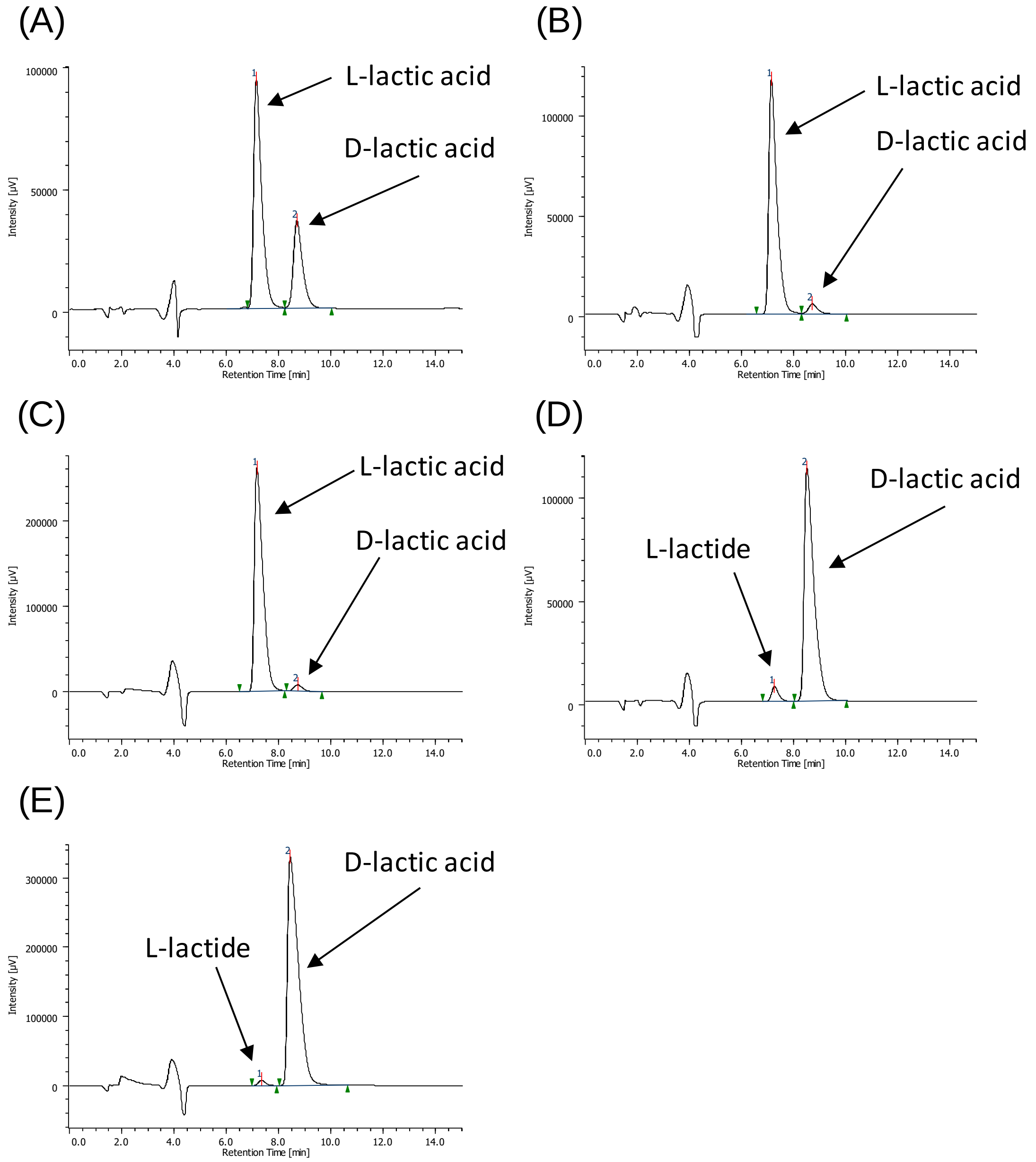

Figure S3. HPLC analyses of lactic acid after hydrolysis of *c*PLA: (A) L-lactide with DMAP in $CHCl_3$, 120 h; (B) L-lactide with DMAP in $scCO_2$, 5 h; (C) L-lactide with DMAP and thiourea in $scCO_2$, 2.5 h; (D) D-lactide with DMAP in $scCO_2$, 5 h; (E) D-lactide with DMAP and thiourea in $scCO_2$, 2.5 h.

3. Preparative GPC to prepare different size of *c*PLA

Three stereocomplex samples with different number-average molecular weights were prepared using *c*PLLA and *c*PDLA to investigate the effect of the ring size on the thermal stability (Table 3 and Figure 4). *c*PLLA and *c*PDLA samples with different number-average molecular weights were isolated by preparative gel permeation chromatography (GPC) using a recycling high performance liquid chromatography (HPLC) system (YMC LC-Forte/R, YMC CO., LTD., Kyoto, Japan) with a YMC-GPC T30000 column (20 × 600 mm) and YMC-GPC T4000 (20 × 600 mm) in series ($CHCl_3$ as the eluent, flow rate 6.0 mL/min).

Table S1. Sample data of *c*PLLA with different ring sizes isolated by preparative GPC.

Sample	Fraction	Mn	Mw	PDI
1	Fr 1-5	9400	11800	1.25
2	Fr 6-7	8500	10300	1.22
3	Fr 8-9	7400	9100	1.23
4	Fr 10-12	6000	7600	1.27
5	Fr 13-15	5300	6700	1.26
6	Fr 16-19	4600	5800	1.25
7	Fr 20-23	4000	5000	1.26
8	Fr 24-28	3300	4100	1.25
9	Fr 29-35	2600	3200	1.20

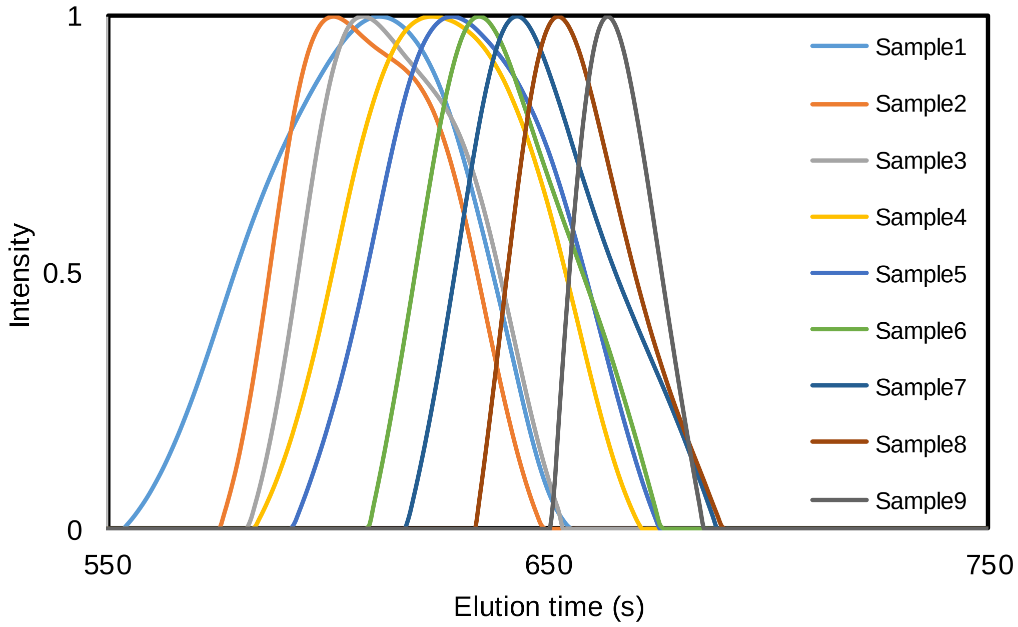

Figure S4. GPC traces of *c*PLLA with different ring sizes isolated by preparative GPC.

Table S2. Sample data of *c*PDLA with different ring sizes isolated by preparative GPC.

Sample	Fraction	Mn	Mw	PDI
1	Fr 1-6	9600	12300	1.29
2	Fr 7-8	7900	9300	1.18
3	Fr 9-11	6800	8100	1.18
4	Fr 12-13	6000	7300	1.22
5	Fr 14-15	5300	6700	1.28
6	Fr 16-17	5100	6300	1.23
7	Fr 18-20	4600	5600	1.23
8	Fr 21-23	3800	4800	1.26
9	Fr 24-27	3300	4200	1.26

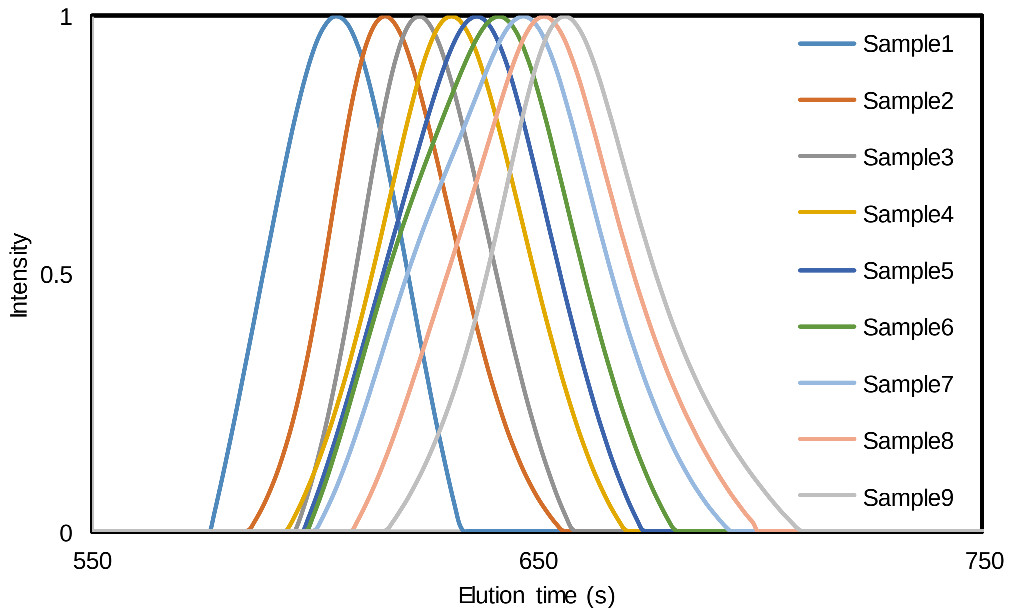

Figure S5. GPC traces of *c*PDLA with different ring sizes isolated by preparative GPC.

4. DSC mesurements

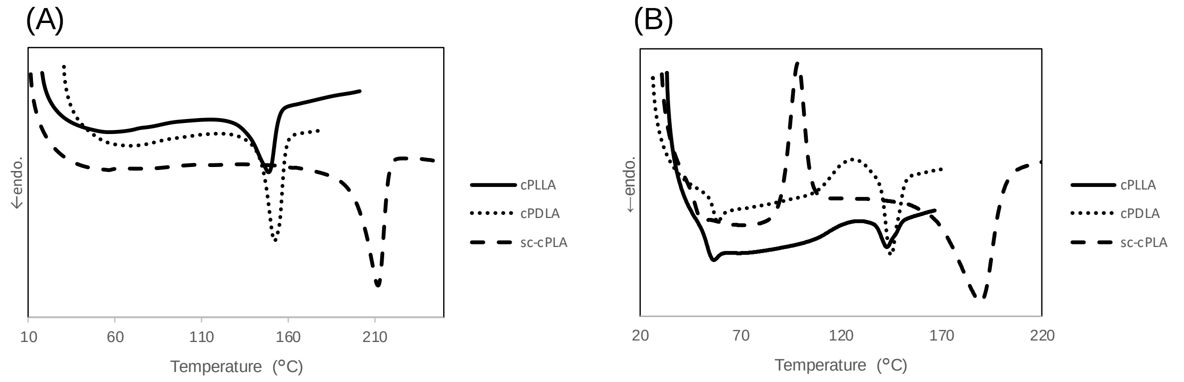

Figure S6. DSC analyses of *c*PLA or sc-*c*PLA: (A) *c*PDLA (Mn = 6,800, PDI = 1.20, 97.0% ee, T_m = 152 °C), *c*PLLA (Mn = 11,000, PDI = 1.60, 94.5% ee, T_m = 149 °C), sc-*c*PLA (T_m = 212 °C); (B) *c*PDLA (Mn = 6,000, PDI = 1.31, 91.0% ee, T_m = 143 °C), *c*PLLA (M_n = 5,500, PDI = 1.40, 90.5% ee, T_m = 145 °C), sc-*c*PLA (T_m = 207 °C).

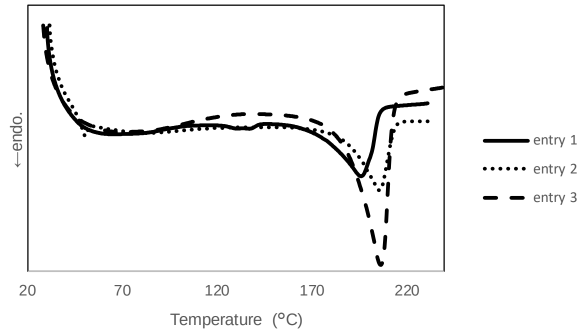

Figure S7. DSC analyses of sc-*c*PLA with different ring size; (entry1) sample *c*PDLA-2 in Table S2 (Mn = 7,900, PDI = 1.18) and sample *c*PLLA-9 in Table S1 (Mn = 2,600, PDI = 1.20), T_m = 190 °C; (entry 2) sample *c*PDLA-6 in Table S2 (Mn = 5,100, PDI = 1.23) and sample *c*PLLA-3 in Table S1 (Mn = 7,400, PDI = 1.23), T_m = 200 °C; (entry 3) *c*PDLA (Mn = 6,000, PDI = 1.31) and *c*PLLA (Mn = 5,500, PDI = 1.40), T_m = 207 °C.
